# Apremilast successfully treats cutaneous polyarteritis nodosa associated with SAPHO syndrome

**DOI:** 10.1093/rheumatology/keae054

**Published:** 2024-06-07

**Authors:** Sandrine Malochet-Guinamand, Angelique Fan, Antoine Perrey, Martin Soubrier

**Affiliations:** Rheumatology Department, CHU Clermont-Ferrand, Clermont-Ferrand, France; Rheumatology Department, CHU Clermont-Ferrand, Clermont-Ferrand, France; Department of Radiology, CHU Clermont-Ferrand, Clermont-Ferrand, France; Rheumatology Department, CHU Clermont-Ferrand, Clermont-Ferrand, France

Rheumatology key message

- Apremilast may be effective for SAPHO syndrome with polyarteritis nodosa.

Dear Editor, Cutaneous polyarteritis nodosa (cPAN) is a rare form of vasculitis affecting small and medium-sized arteries in the dermis and subcutaneous tissue. It lacks standardized treatment [1]. Synovitis, acne, pustulosis, hyperostosis and osteitis (SAPHO) syndrome is a rare autoinflammatory disorder characterized by cutaneous and osteoarticular manifestations [2]. Due to the rarity of these two conditions, current evidence-based management is limited to case reports. The association of these two entities has never been described in the literature. Here we present a unique clinical case of a patient with skin lesions and bone pain. Written informed consent for publication was obtained from the patient.

In 2020, a 53-year-old man with no significant medical history developed pain initially localized to the right anterior tibial tuberosity, which later extended to the entire tibia, including the contralateral tibia. Subsequently, he developed large infiltrative skin lesions, specifically livedoid plaques and nodules (Fig. 1A). Initially, tumour lesions were suspected. In November 2020, whole-body bone scan showed increased activity in the sternoclavicular joints, both upper tibiae and the right tibial diaphysis, with particularly heightened fixation in the right medial malleolus (Fig. 1B). CT scan of the upper right tibia showed a small reaction of osteitis at the anterior tibial tuberosity. (Fig. 1C). MRI showed clear osteitis of the anterior tuberosity of the right tibia with florid hyperostosis of the periosteum and inflammation of the surrounding soft tissues (Fig. 1D).

**Figure 1.**
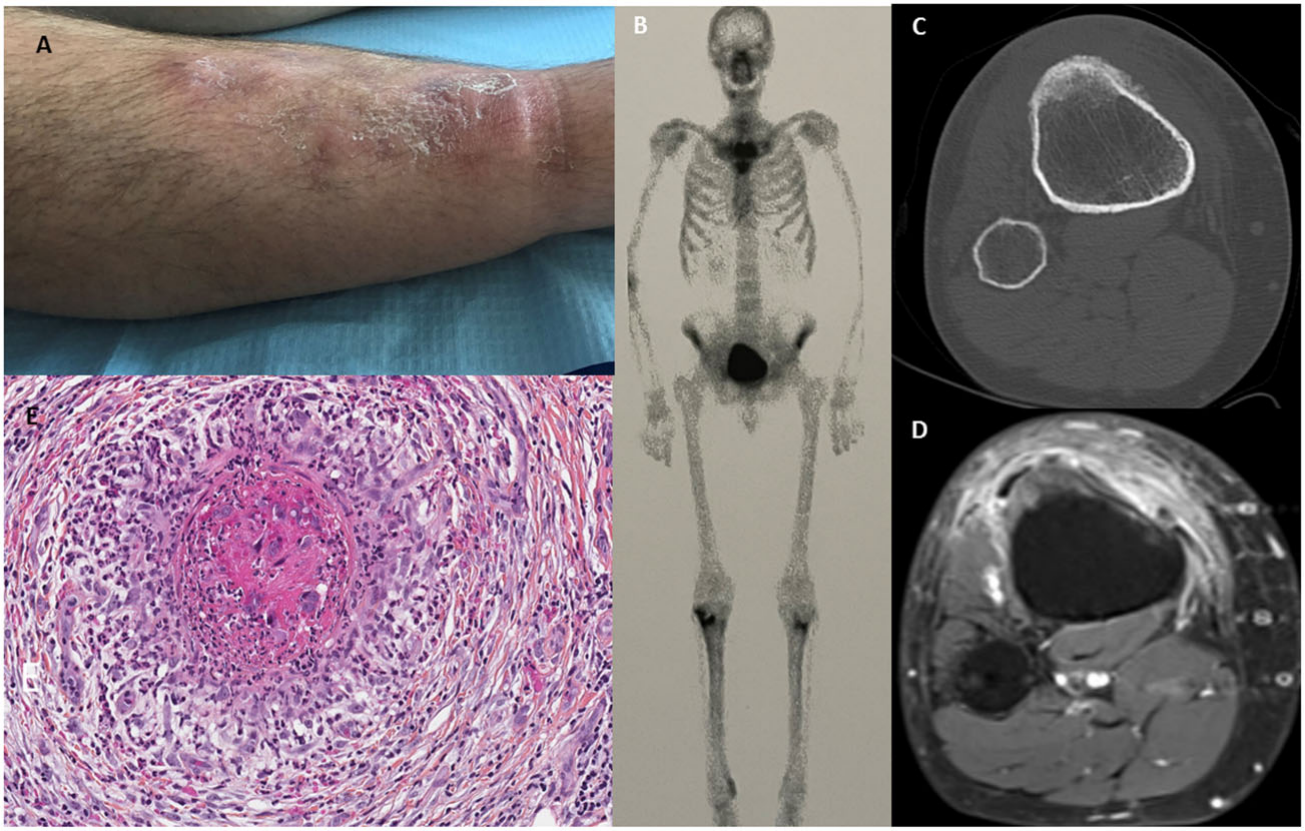
Diagnostic clinical examination. (**A**) Right lower leg skin lesions. (**B**) Whole-body bone scan demonstrated lesions in bilateral sternoclavicular joints, bilateral upper tibiae and tibial diaphysis, and in the right medial malleolus. (**C**) Axial CT scan of the right upper tibia showing hyperostosis and a small reaction of osteitis at the anterior tibial tuberosity. (**D**) MRI–Axial T1 fat saturated post contrast image at the level of the anterior tibial tuberosity with hyperostosis and clear osteitis. (**E**) Histological examination showing vasculitis with arterial luminal thrombosis and variable parietal inflammatory infiltrates of neutrophils

In February 2021, an initial skin biopsy was in favour of hypodermic neutrophil collection. The patient has swelling of the right ankle. Full blood count was normal, viral serologies, RF, CCP antibody, ANA and ANCA were negative, and ESR was within normal limits. The level of CRP (32.2 mg/l) and haptoglobine (3.16 g/l) were elevated. Our investigations ruled out systemic involvement.

In April, a recurrence of skin lesions on the leg with nocturnal pruritus led to a new deep biopsy showing necrotizing vasculitis small vessels in the hypodermis (Fig. 1E). The diagnosis was SAPHO syndrome associated with cutaneous periarteritis nodosa. The patient initially received treatment with pamidronate in July, which improved his skin condition but exacerbated his bone pain. The pain was inflammatory, bilateral and predominantly in the right proximal lower extremity and ankle. The patient was therefore switched to apremilast 30 mg twice daily in January 2022. The tibial pain improved rapidly, but significant side effects such as diarrhoea, epigastric pain and nausea were observed. Therefore, after three months of treatment with apremilast, the dose was reduced to 30 mg per day. Within six months, the patient experienced complete resolution of skin manifestations and significant improvement in bone pain. At 18 months, the disease remains stable.

To the best of our knowledge, this is the first report of cPAN associated with SAPHO. This rare association presents additional diagnostic and therapeutic challenges. [1, 3]. Apremilast is FDA-approved for the treatment of psoriasis and psoriatic arthritis and is used off-label for various dermatological conditions resistant to or ineffective with conventional therapy. Apremilast’s efficacy in vasculitis has never been tested, and its effectiveness in SAPHO has only been documented in one case by Adamo *et al.* [4]. This case presented with cutaneous manifestations including palmoplantar pustulosis and sternoclavicular joint pain and had failed multiple conventional synthetic and biologic treatments. Switching to apremilast after failure of ustekinumab, adalimumab and secukinumab results in stabilisation of skin and joint symptoms without side effects. Our patient’s initial biopsy was negative because multiple stages can coexist in the same artery. This highlights the importance of multiple biopsies, serial sections and longitudinal section analysis before excluding vasculitis [5]. In conclusion, this case illustrates the unusual combination of cPAN and SAPHO syndrome and suggests the potential therapeutic role of apremilast in managing these two conditions.

## Data Availability

Data will be shared upon reasonable requests to the corresponding author.
